# Biomarkers in breast cancer: A consensus statement by the Spanish Society of Medical Oncology and the Spanish Society of Pathology

**DOI:** 10.1007/s12094-017-1800-5

**Published:** 2017-12-22

**Authors:** R. Colomer, I. Aranda-López, J. Albanell, T. García-Caballero, E. Ciruelos, M. Á. López-García, J. Cortés, F. Rojo, M. Martín, J. Palacios-Calvo

**Affiliations:** 1 Departamento de Oncología Médica, Hospital Universitario La Princesa, C/Diego de León, 62, 28006 Madrid, Spain; 2 Pathology Department, General University Hospital of Alicante, Alicante, Spain; 3 Medical Oncology Department, Mar University Hospital, Hospital del Mar Medical Research Institute (IMIM), Pompeu Fabra University, CIBERONC, Barcelona, Spain; 4 Pathology Department, University Hospital Complex of Santiago, Santiago de Compostela, Spain; 5 Medical Oncology Department, Doce de Octubre University Hospital, Madrid, Spain; 6 Pathology Department, Virgen del Rocio University Hospital, CIBERONC, Seville, Spain; 7 Medical Oncology Department, Ramón y Cajal University Hospital, Madrid, Spain; 8 Vall d’Hebron Institute of Oncology (VHIO), Barcelona, Spain; 9 Baselga Institute of Oncology (IOB), Madrid, Barcelona Spain; 10 Pathology Department, Fundación Jiménez Díaz University Hospital, Madrid, Spain; 11 Medical Oncology Department, Gregorio Marañón University Hospital, CIBERONC, GEICAM, Madrid, Spain; 12 Pathology Department, Ramón y Cajal University Hospital, CIBERONC, IRYCIS and University of Alcalá, Madrid, Spain

**Keywords:** Breast neoplasm, Diagnostic, Gene expression profiling, Prognostic, Therapy predictive

## Abstract

This consensus statement revises and updates the recommendations for biomarkers use in the diagnosis and treatment of breast cancer, and is a joint initiative of the Spanish Society of Medical Oncology and the Spanish Society of Pathology. This expert group recommends determining in all cases of breast cancer the histologic grade and the alpha-estrogen receptor (ER), progesterone receptor, Ki-67 and HER2 status, in order to assist prognosis and establish therapeutic options, including hormone therapy, chemotherapy and anti-HER2 therapy. One of the four available genetic prognostic platforms (MammaPrint^®^, Oncotype DX^®^, Prosigna^®^ or EndoPredict^®^) may be used in node-negative ER-positive patients to establish a prognostic category and decide with the patient whether adjuvant treatment may be limited to hormonal therapy. Newer technologies including next-generation sequencing, liquid biopsy, tumour-infiltrating lymphocytes or PD-1 determination are at this point investigational.

## Introduction

Biomarker analysis in cancer not only provides additional information about classical clinical factors, but also enables patients with a more favourable benefit–risk balance to receive certain treatments [1]. In breast cancer, biomarker analysis is routine practice. It originally began with testing for hormone receptor expression to guide tamoxifen therapy. The subsequent inclusion of targeted treatments against human epidermal growth factor receptor 2 (HER2) revolutionised the biomarker field. It also demonstrated that biomarker test methods needed to be standardised and harmonised. Recognising that need, scientific societies in several countries have written and published consensus guidelines. Among these were the first guidelines on recommendations for HER2 testing in breast cancer put forward by the Spanish Society of Pathology (SEAP) and the Spanish Society of Medical Oncology (SEOM) in 2009 [2, 3]. Since then, the main change in the management of infiltrating breast carcinoma in terms of biomarker testing has been the inclusion of genetic platforms. These were initially designed to assist prognosis and to predict chemotherapy response in patients with tumours that express hormone receptors, and no lymph-node metastases. The intervening years have also seen progress in the understanding of molecular abnormalities in breast cancer from studies using next-generation sequencing techniques. The clinical potential for monitoring disease using new technologies grouped under the term “liquid biopsy” is currently being studied. Also, as with other cancers, there is growing interest in knowing what impact immunotherapy and related biomarker testing will have on the future management of breast cancer patients.

The purpose of these SEOM–SEAP consensus guidelines is to recommend which biomarkers should routinely be tested in patients with breast cancer, including conventional markers, genetic platforms and newer technologies.

## Testing conventional and non-conventional markers

### Histological grade

Histological grade is a parameter that has independent prognostic value at all stages of breast cancer that adds to axillary status and tumour size. All invasive breast carcinomas should therefore be graded [4, 5]. The combined histological grade simply and efficiently provides biological information about the tumour, directly related to proliferation (mitosis), abnormal architecture, nuclear shift, and the expression of chromosomal instability [4]. The World Health Organization (WHO) classification and the College of American Pathologists (CAP) guidelines recommend using the Nottingham (Elston–Ellis) modification of the Patey–Scarff and Bloom–Richardson grading system [6, 7]. The inter-observer agreement level is very high when these recommendations are strictly followed. Also, they can be applied to tissue obtained by core-needle biopsy (CNB) [8].

### Estrogen receptor and progesterone receptor

Expression of estrogen receptor (ER)-alpha is a favourable prognostic factor and strongly predictive of a response to hormone therapy [9]. Approximately 30–40% of patients with ER-expressing advanced breast cancer will have an objective response to hormone treatment, and a further 20% of patients will achieve disease stabilisation. Moreover, the hormone therapy response in patients with early ER-expressing breast cancer, in terms of overall and disease-free survival, is well known [10, 11]. Hormone therapy is relatively non-toxic. Its long-lasting clinical activity justifies its use in any patient with an ER-expressing mammary tumour.

The technique used to test for ER can be applied inexpensively to fixed, paraffin-embedded tissue. It is therefore readily available in most Pathology Departments. Examining tissue under the microscope means that positive reactions can be assessed in tumour cells only, avoiding problems with low cell density or normal breast tissue included in the tumour growth. Detailed guidelines addressing methods for the immunohistochemical analysis of ERs and progesterone receptors (PRs) are available [12, 13].

In general, 70–75% of invasive breast carcinomas express ER-alpha. A positive reaction is seen in the nucleus. Staining intensity and the percentage of positive cells can vary. The morphological context should be taken into account. In apparently negative cases of certain special histological types, such as tubular, mucinous or lobular carcinoma, or in histological grade I, confirmation of the results should be considered. The cut-off point for defining a positive result is ≥ 1% of nuclei positive, irrespective of staining intensity. The reported results should include the antibody clone used. It is advisable to include the percentage of positive cells. Alternatively, a score can be reported, like the one described by Allred et al., combining the estimated nuclear positivity rate in cancer cells (a score of 0–5, based on the percentage) with staining intensity (intensity 0–3) [14]. It is also useful to test for ER-alpha in ductal carcinoma in situ, because hormone suppression treatment can reduce the recurrence risk by 50% in patients expressing this receptor.

PRs are regulated by ER-alpha, so expression of PRs suggests that the oestrogen/ER-alpha pathway is functional. As with ER-alpha, biochemical methods to test for PR expression were replaced in the 1990s by immunohistochemistry, which is the recommended technique [12, 13]. PRs are expressed in 60–70% of cases of invasive ductal carcinoma of the breast. In general, correlation between ER-alpha and PR expression is good, although 10% of cases may prove to be ER-alpha-positive and PR-negative. These patients have a higher risk of recurrence than ER-alpha-positive, PR-positive cases. Fewer than 5% of patients may prove to be PR-positive, ER-alpha-negative. Their prognosis is similar to that of ER-alpha-positive, PR-positive patients. The methodology and quantification used are the same as for ER-alpha, with positive cases usually defined as 1% or more. Some recent studies suggest that low-level PR expression (< 20%) might have negative prognostic implications. Including it as one of the parameters for distinguishing the Luminal subtype has therefore been suggested [15, 16].

### Ki-67

Immunohistochemical assessment of Ki-67 is the method most widely used in clinical practice to determine the proliferative activity of breast cancer. Ki-67 is particularly important for distinguishing risk groups in carcinomas positive for ER-alpha and PR. The available guidelines on Ki-67 assessment in breast cancer address methodological issues in the various phases [17]. Calibrating the method in different laboratories substantially increases the concordance between results [18]. There is no absolute agreement regarding cut-off points. It has been recommended that each pathology department should set its most appropriate cut-off points [17]. Some guidelines define “low proliferative activity” as Ki-67 levels below 10%, and “high proliferative activity” as levels above 30%. However, the critical point is usually between 10 and 20% [18].

In combination with PR expression levels, the St Gallen consensus established four categories based on Ki-67 levels: < 14, 14–19, 20–25 and > 25%. A 20% cut-off was recommended for distinguishing between Luminal A-like and Luminal B-like tumour types [19]. A recent meta-analysis concluded that a Ki-67 level of over 25% is associated with a worse prognosis [20].

Ki-67 quantification appears to have clinical applicability in the choice of adjuvant therapy for ER-expressing tumours. In combination with other clinical factors, its validity is comparable to that of more complex gene expression analyses [21]. However, American Society of Clinical Oncology (ASCO) guidelines on using biomarkers to guide decisions on adjuvant therapy do not recommend its use [22]. More international studies of a collaborative nature are needed, to standardise values of this marker so that it can be clinically validated [23].

### HER2

Along with hormone receptors, HER2 is the most important prognostic and predictive marker in breast cancer. Since the early studies by Slamon in 1987, it has been known that breast cancers that overexpress HER2 represent a highly aggressive biological subtype [24]. However, the 1998 approval of trastuzumab for therapeutic use changed the outcome in these patients, whose clinical course improved very significantly. The introduction of new targeted anti-HER2 therapies, such as lapatinib, pertuzumab and trastuzumab emtansine (T-DM1), the last one administered with no requirement for simultaneous cytostatics, underlines the importance of identifying patients with HER2-positive breast cancer.

**Fig. 1:**
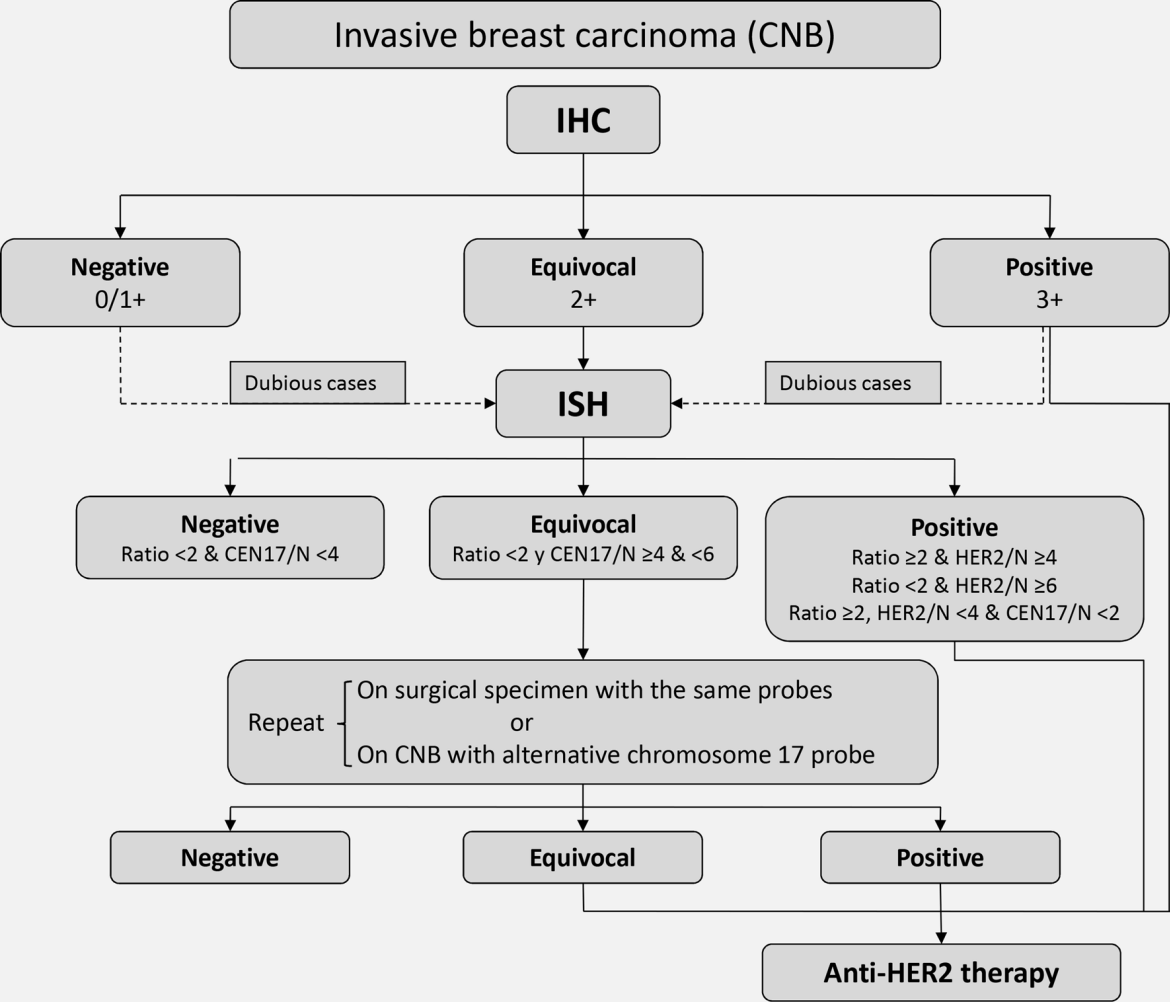
Algorithm for HER2 testing on a sample of invasive breast carcinoma obtained by core-needle biopsy. *HER2* human epidermal growth factor receptor 2, *IHC* immunohistochemistry, *ISH* in situ hybridisation

Any invasive breast carcinoma should be tested for HER2 overexpression, along with ERs, PRs and Ki-67. A CNB sample is sufficient, and in most cases the test does not need to be repeated on material from the surgical specimen (Fig. 1). Fixation time is much more standardised for CNBs (normally 6–24 h) than for surgical specimens, and concordance between the two tests is very high (98–99%) [25, 26]. Using CNB material also means that the information is available for clinicians before making a decision about possible neoadjuvant therapies. This test is performed by immunohistochemistry and/or in situ hybridisation (ISH), fluorescence in situ hybridisation (FISH) or chromogenic in situ hybridisation (CISH or SISH).

Various guidelines conclude that any HER2 test method is valid, provided the technology is standardised according to the manufacturer’s instructions, and supported by an external quality-control programme. This tends to be routine practice in pathology laboratories across Spain [2, 27]. In order to ensure high-quality testing, it is very important for the number of technicians who perform the test, and especially the number of pathologists who interpret the results, to be as low as possible [2]. Immunohistochemistry is the most widely used technique for HER2 expression status analysis. Not only is it available in all pathology laboratories, but also it allows the sample to be assessed cheaply, simply and quickly. In addition, it provides an overview of the sample, permitting easy identification of possible small positive foci in heterogeneous cases.

Results should be interpreted according to the recommendations in the ASCO/CAP guidelines [27]. The main change introduced by the current guidelines was the additional inclusion of incomplete membrane staining in the definition of equivocal cases (2 +). This change has been criticised for entailing an unnecessary increase in ISH tests [28]. The rationale for introducing it was mainly based on micropapillary carcinomas, which often show moderate-to-intense lateral or basolateral staining and can display amplification [29]. The new version of the ASCO/CAP guidelines currently being prepared is likely to revert to the previous definition of 2 +, which required complete membrane staining [29]. At the moment, heterogeneous cases that are mostly negative but have a small focus (≤ 10% of cells) of complete, intense, positive staining are also classified as 2 +. ISH is recommended for cases that are equivocal (2 +) or indeterminate (cannot be assessed for technical reasons), and also in all cases of dubious interpretation (1 + versus 2 + or 3 + versus 2 +).

Whether tests should be repeated because of histological discordance is also debatable. This recommendation was introduced in the 2013 guidelines. In particular, it seems unnecessary to retest grade 3, HER2-negative cases [28, 29], or to repeat tests on the surgical specimen when CNB results were negative [29]. In the case of available metastatic, however, the test may be repeated due to the possibility of cells becoming positive, because it is very rare for them to become negative. In biopsies of bone metastases, the process of decalcifying the material generally prevents reliable HER2 testing, and can lead to false negatives. Even so, if a mass is available, the test can safely be done. The arrival of a new generation of weaker decalcifiers, better at preserving both antigens and nucleic acids, might solve this problem in the future.

The technique of ISH complements immunohistochemistry. It has several advantages: it is little affected by fixation; results are read objectively by counting signals; and normal cells and tumour cells provide a positive internal control. However, it is slower to read than immunohistochemistry because of the counting procedure required, and small foci of amplification can more easily be missed. In the current ASCO/CAP guidelines, the threshold for the HER2/CEN17 ratio was simplified back to the original 2.0, in addition to taking account of the number of HER2 signals per cell [27]. Thus, even with a negative ratio, if the number of HER2 signals per cell is 6 or more, the result is positive; and if it is between 4 and 6, the result is equivocal and the test must be repeated. This can either be done on the same sample, using an alternative chromosome 17 probe such as *RARA* or *TP53*, or the test can be repeated on material from the surgical specimen. If the result is still equivocal after repetition, the oncologist may consider prescribing an anti-HER2 therapy, which is normally done to minimise risks [27]. It is important for future guidelines to avoid these ambiguities so that pathologists’ reports are conclusive for therapy. Analysis of the response obtained in large series of patients with polysomy, following treatment of equivocal cases, might provide valuable data on this issue. In fact, in the draft of the new version of the ASCO/CAP guidelines, if the specimen test result is equivocal both by IHC and FISH, it is recommended that the sample be considered HER2 negative.

Heterogeneous amplification, although uncommon in breast carcinomas compared with gastric tumours, often raises doubts about quantification. If a cohesive amplified clonal focus is seen, only that clone should be counted, with a generally positive result. If, in contrast, amplified cells appear mingled with unamplified cells (“salt and pepper”) they should all be counted and reported in terms of the means obtained, and the percentage of amplified cells should also be stated [30].

## Prognostic genetic platforms: molecular phenotypes and translation to the clinic

In the last few years, clinical practice in Spain has witnessed the arrival of four genetic platforms for determining the prognosis of patients with ER-positive, HER2-negative tumours of favourable prognosis, without lymph nodes involved. All these platforms are used to evaluate the risk of recurrence. However, they differ substantially in the methodology used to quantify gene expression, the genes tested, the clinical and pathological variables included, risk group stratification, and whether or not testing takes place in centralised laboratories. It should therefore come as no surprise that, although they are all of proven clinical usefulness and analytically validated, results from the various platforms can place the same patient into different risk categories.

Cost-effectiveness analyses have suggested that the use of genetic platforms is cost effective in that it reduces chemotherapy use, and prevents the occurrence of events during the clinical course [31–35], although the current use of generic chemotherapy drugs poses some doubts about the actual economic impact. The various European and American clinical guidelines, and several expert groups, make a range of recommendations for using genetic platforms in different clinical contexts in hormone-dependent breast cancer, either as a prognostic tool or to establish the benefit of supplementing hormone therapy with chemotherapy [22, 36–40] (Tables 1, 2). A very comprehensive scientific review, that includes economic implications of the platforms, has been very recently been published [41].

**Table 1:** Usage recommendations for different genetic tests as prognostic tools or to establish the benefit of adding chemotherapy to hormone therapy in the management of breast cancer

	Oncotype DX^®^	MammaPrint^®^	Prosigna^®^ (PAM50)	EndoPredict^®^
ASCO	Guides the decision to prescribe adjuvant systemic chemotherapy Evidence: high Recommendation: strong	Should not be used for decision-making about adjuvant systemic chemotherapy use Evidence: intermediate Recommendation: moderate	Guides the decision to prescribe adjuvant systemic chemotherapy together with other clinical and pathological variables Evidence: high Recommendation: strong	Guides the decision to prescribe adjuvant systemic chemotherapy Evidence: intermediate Recommendation: moderate
NCCN	The only test recommended for patients with > 0.5 cm tumour Oncotype DX^®^: can be considered for selecting patients with 1–3 ipsilateral lymph nodes involved The only test validated for predicting chemotherapy response	Prognostic value, but not validated for predicting chemotherapy response		
St Gallen 2015	Prognostic value and predictive of the benefit of adjuvant chemotherapy			
SEOM	5-year recurrence risk prognosis: IA/IB 10-year recurrence risk prognosis: IB Chemotherapy benefit prediction: IA/IB	5-year recurrence risk prognosis: IB 10-year recurrence risk prognosis: – Chemotherapy benefit prediction: –	5-year recurrence risk prognosis: IB 10-year recurrence risk prognosis: IB Chemotherapy benefit prediction: –	5-year recurrence risk prognosis: IB 10-year recurrence risk prognosis: IB Chemotherapy benefit prediction: –
IMPAKT	Little but significant prognostic information above and beyond clinical and pathological parameters. No evidence of clinical usefulness for modifying the treatment decision			

*ASCO* American Society of Clinical Oncology, *IMPAKT* Improving Care and Knowledge Through Translational Research in Breast Cancer, *NCCN* National Comprehensive Cancer Network, *SEOM* Spanish Society of Medical Oncology

**Table 2:** Prognostic and predictive value of different genetic tests in breast cancer

	ASCO 2016			NCCN 2016		ESMO 2015			SEOM 2015		
	Prognosis		CT benefit prediction	Prognosis	CT benefit prediction	Prognosis		CT benefit prediction	Prognosis		CT benefit prediction
	5 years	10 years				5 years	10 years		5 years	10 years	
Oncotype DX^®^	Yes	NA	Yes	Yes	Yes	+++	+++	Yes	IA (low RS) IB (other RSs)	IB	IA (low RS) IB (other RSs)
Prosigna^®^	Yes	Yes	Yes	Yes	NA	++	++	Yes	IB	IB	NA
MammaPrint^®^	Yes	–	–	Yes	NA	+++	NA	Yes	IB	NA	NA
EndoPredict^®^	Yes	Yes	Yes	Yes	NA	++	++	Yes	IB	IB	NA

*ASCO* American Society of Clinical Oncology, *CT* chemotherapy, *ESMO* European Society for Medical Oncology, *NA* not available, *NCCN* National Comprehensive Cancer Network, *RS* Recurrence Score, *SEOM* Spanish Society of Medical Oncology

### MammaPrint^®^

The MammaPrint^®^ 70-gene expression platform yields a signature that divides breast carcinomas into two risk categories, i.e. high and low [42].

The platform has been validated in several studies, and provides prognostic information for distant disease-free survival independently of the usual clinical and pathological criteria [43–45].

In 2007, the platform was approved by the Food and Drug Administration (FDA) for determining prognosis in patients aged 60 years or under with node-negative, stage I–II tumours measuring ≤ 5 cm. In 2009, it obtained a second approval for patients over 60 years old. More recently, MammaPrint^®^ has been validated for paraffin-embedded material [46].

Various studies have indicated its prognostic value for determining 10-year distant metastasis-free survival in patients with breast cancer involving 1–3 axillary lymph nodes, in women at low risk, and for HER2-positive tumours [47, 48]. It has also been shown that MammaPrint^®^ is useful for establishing the benefit of administering chemotherapy [49, 50].

The MINDACT trial (Microarray in Node 0–3 positive Disease may Avoid Chemotherapy, EORTC10041/BIG03/04, NCT00433589) was a multicentre, prospective, randomised, Phase III study involving over 6000 patients. It demonstrated that, in 1550 cases of high clinical risk but low genomic risk, 5-year metastasis-free survival was 94%, suggesting that approximately 46% of high-risk cases might not need chemotherapy (level of evidence IA) [51–53].

The 2017 update of the ASCO Clinical Practice Guideline of Biomarkers use for the adjuvant therapy of breast cancer, focused on the use of MammaPrint^®^, specified that MammaPrint^®^ may be used in patients with HR+, HER2-negative cases with 1–3 positive nodes AND a high clinical risk to inform decisions on withholding adjuvant chemotherapy. The ASCO guideline warns that these patients should be informed that a benefit of chemotherapy cannot be excluded, particularly in patients with ≥ 1 nodes involved. On the other hand, MammaPrint^®^ does not have a use in the low-risk category nor in patients with HER2+ or triple-negative breast cancer, according to the guideline [54].

### Oncotype DX^®^

Oncotype DX^®^ tests the expression of 21 genes (16 cancer-related genes and 5 reference genes) and calculates a Recurrence Score (RS) [55, 56].

Oncotype DX^®^ methodology has been optimised for application to formalin-fixed tissue, and its results have a proven impact on treatment decisions [57–60].

The RS defines three groups: low RS with a value under 18; intermediate RS from 18 to 30; and high RS with values of 31 or over. Several studies have shown that the 10-year distant recurrence rate is 7% in the low RS group, 14% in the intermediate RS group, and 30% in high RS patients [56, 61].

The value of Oncotype DX^®^ for predicting the benefit provided by chemotherapy and hormone therapy in these risk groups has been examined in various studies, involving both node-negative and node-positive patients [62–64], although the 2016 ASCO Guideline recommends the use of Oncotype to guide decisions about adjuvant chemotherapy only in cases without lymph node involvement [22].

Oncotype DX^®^ has been shown to provide information above and beyond the clinical and pathological features in postmenopausal patients with hormone-dependent breast cancer treated with an aromatase inhibitor.

TAILORx (Trial Assigning Individualized Options for Treatment [Rx]) was a prospective trial designed to determine the prognosis of a group of patients who had undergone surgery for ER-positive, HER2-negative, node-negative breast cancer, with an RS of 11–25 [65]. Recently published results from the RS < 11 group reported a distant recurrence risk of 0.7%, and a 1.3% risk of any other recurrence. These results were confirmed in the Surveillance, Epidemiology and End Results (SEER) database registry [66].

Lastly, the RxPONDER study (Rx for Positive NoDe, Endocrine Responsive Breast Cancer) will prospectively report the benefit of chemotherapy in women with low RS and involvement of 1–3 axillary lymph nodes.

### Prosigna^®^

The Prosigna^®^ test is a genomic classifier based on a 50-gene signature (PAM50). It was initially designed using RT-qPCR on paraffin-embedded tissue [67, 68]. This test can be carried out in decentralised laboratories [69].

Prosigna^®^ provides information on the intrinsic tumour subtype (Luminal A, Luminal B, HER2-enriched or basal-like). It also determines the 10-year risk of distant recurrence, as a Risk of Recurrence (ROR) score on a scale of 0–100. Scores are categorised as low (ROR score < 40, less than 10% risk), intermediate (ROR score 40–60, 10–20% risk), or high (ROR score > 60, over 20% risk of recurrence).

The clinical validity of Prosigna^®^ has been tested in several studies. These studies have demonstrated that the ROR score provides prognostic information above and beyond the standard clinical and pathological variables [70, 71], with level IB evidence [72]. Moreover, the ROR score is significantly correlated with distant metastasis-free survival, and adds medium- and long-term prognostic information (more than 10 years). It has also been confirmed that Prosigna^®^ provides prognostic information about recurrence after 10 years of hormone therapy.

The impact of Prosigna^®^ on therapeutic decision-making has also been demonstrated [73]. Prosigna^®^ has obtained the CE mark in Europe, FDA accreditation, and approval by Health Canada for predicting 10-year distant recurrence in postmenopausal women with 1–3 axillary lymph nodes involved.

### EndoPredict^®^

EndoPredict^®^ is another second-generation genomic classifier, based on testing 12 genes by RT-PCR on paraffin-embedded tissue: 8 cancer genes, 3 reference genes for standardisation, and one for measuring genomic DNA [74, 75]. It is a decentralised test that can be carried out in any laboratory. The clinical validity of EndoPredict^®^ for predicting distant recurrence independently of the classical clinical and pathological parameters was confirmed in two clinical trials evaluating adjuvant hormone treatment (ABCSG-6 and ABCSG-8). It was also validated in a study by the GEICAM group, in node-positive women treated with adjuvant hormone therapy and chemotherapy. It therefore possesses type IB evidence for prognosis [76, 77].

EndoPredict^®^ provides information on the distant recurrence risk according to gene expression (genomic EP score [EP]), and the risk adjusted for tumour size and number of lymph nodes involved (clinical EP score [EPclin]). On a scale of 0–15, it defines two categories based on the 10-year distant recurrence risk: low risk (score < 3.4; overall risk of 6–8%) and high risk (score > 3.4; overall risk of 15–22%).

EndoPredict^®^ has been awarded European certification for clinical use (CE mark for IVD).

## New technologies

### Next-generation sequencing

Different next-generation sequencing (NGS) studies [78–83] have demonstrated that the most frequently mutated genes in breast cancer are *PIK3CA* (31–41%), *TP53* (30–36%), *KTMC2* (7–11%), *GATA3* (10–11%), *MAP3K1* (7–10%), and *CDH1* (10–11%). Whereas *MAP3K1/TP53*, *GATA3/TP53*, *CDH1/TP53*, and *CDH1/GATA3* mutations were mutually exclusive, concomitant mutations of *MAP3K1/PIK3CA*, *CDH1/PIK3CA* were frequently observed.

Regarding intrinsic breast cancer subtypes, mutations in *PIK3CA* were observed in 43–57% of Luminal A and in 31–35% of Luminal B carcinomas, respectively. The most important difference between both types of tumours was the frequency of *TP53* mutations, which was 11–12% in Luminal A, but 24–29% in Luminal B breast carcinomas. *TP53* and *PIK3CA* mutations have been detected in approximately 70 and 40% of HER2-enriched breast carcinomas and in 89 and 16% of basal breast carcinomas, respectively [83].

Regarding NGS studies in specific histological subtypes, Ciriello et al. profiled 127 invasive lobular carcinomas (ILC) and compared the distribution of mutations with a subset of infiltrative ductal carcinomas (IDC), particularly with Luminal A, given that 83% of ILC are classified as Luminal A by PAM50 [84]. The most frequent mutations were detected in *CDH1* (65%), *PIK3CA* (48%), *RUNX1* (10%), *TBX3* (9%), *PTEN* (8%), *TP53* (8%), *FOXA1* (7%), *MAP3K1* (6%), and *GATA3* (5%). In addition to *CDH1* loss, the molecular hallmark of ILC, ILC and IDC differed in the *FOXA1*, *GATA3* and *TBX3*, *PTEN* loss and AKT activation. The lower incidence of *GATA3* and the higher incidence of *FOXA1* mutations in ILC, and their roles as regulators of ER activity, suggest that *GATA3* and *FOXA1* regulate the ER receptor by alternative mechanisms. ILC has the highest levels of *AKT* activation comparable to basal IDC, making selective inhibition of this pathway a hypothetical therapeutic strategy in these tumours. Finally, 14% of ILC showed *PTEN* inactivation, compared to 3% of IDC, either by homozygous deletions or mutations, and were mutually exclusive with *PIK3CA*. Similar results have been subsequently reported by Desmedt et al.

At present, NGS in breast cancer remains a research tool. A recent consensus group has suggested that for selecting breast cancer patients for clinical trials investigating new drugs, an optimal gene panel should detect *AKT1*, *PIK3CA*, *PTEN*, *ESR1* mutations and *FGFR1* amplification, in addition to the study of ER, PR, HER2 and BRCA1/2 [85].

### Liquid biopsy and circulating tumour cells

Liquid biopsies, defined broadly as either circulating tumour cells (CTCs) of epithelial origin, tumour nucleic acids (ctDNA, cfmiRNA), or tumour exosomes in the blood of cancer patients, have received increasing attention as a new diagnostic tool. To date, diagnosis and metastasis monitoring is mainly carried out through tissue biopsy and/or re-biopsy, an invasive procedure limited only to certain locations and not always feasible in clinical practice. In order to improve tumour characterisation and disease monitoring over time, liquid biopsy may represent a new tool. Technologies for detecting and isolating CTCs include the FDA-validated CellSearch^®^ system, but other technologies are gaining prominence [86].

CTCs have been proved to be a significant prognostic factor in both early and metastatic breast cancer [87]. In fact, CTC positivity constitutes an individual risk factor for breast cancer relapse/death not inferior to the usual prognostic factors (size, grade, proliferation or node status) that are currently taken into account for adjuvant treatment decision [88]. However, no definitive evidence supports its clinical utility at the moment. As opposed to CTCs enumeration, molecular characterisation of the CTCs might potentially be helpful as a predictive biomarker for therapy selection [89, 90].

Emerging data support a potential role of ctDNA in breast cancer. In a study performed in patients with early breast cancer treated with neoadjuvant chemotherapy, the detection of ctDNA post-surgery or during follow-up was highly predictive of relapse, resistance to therapy, and prediction of response. Another potential use of ctDNA is to detect *ESR1* mutations, which predict resistance to aromatase inhibitors (but not fulvestrant) in advanced ER-positive breast cancer or *PI3K* mutations, which may predict the benefit of some PI3K inhibitors; these are under development [91, 92].

The use of NGS in liquid biopsy may further improve our ability to predict relapse, monitor patients, predict drug activity, or provide early detection of resistance.

### Tumour-infiltrating lymphocytes

In the last few years, morphological evaluation of tumour-infiltrating lymphocytes (TILs) in breast cancer has been proposed as a potentially useful biomarker given the prognostic value observed in triple-negative breast cancer (TNBC) [93, 94], and HER2 subtypes [93, 95]. It has been reported that every 10% increment of stromal lymphocytes was associated with an 18% reduction of risk of death [94, 95].

However, the majority of panelists of the 2017 St Gallen Consensus Conference did not recommend using TILs as a new prognostic factor in TNBC patients, in view of the absence of standardised guidelines for their evaluation, data on methodological reproducibility, or clinical validation [37]. The International TILs Working Group experienced in TILs evaluations recently issued recommendations for harmonising and improving consistency in scoring TILs, including detailed guidelines for annotating the prevalence of lymphocyte infiltration, which may minimise inter-observer reproducibility [96] (Table 3). In order to evaluate the feasibility and utility of these recommendations in clinical practice, Prunery et al. carried out a retrospective analysis of a series of 897 patients with TNBC [97]. Multivariable analysis confirmed, in agreement with previous studies, that each 10% increase in TILs strongly predicted better survival independent of patients’ age, lymph node status, histological grade, peritumoural vascular invasion, and Ki-67 labelling index. Stratified analysis revealed a positive correlation between TILs and overall survival across all the subgroups analysed.

**Table 3:** Recommendations of the TILs Working Group’s for assessing TILs in breast (for further detail see Salgado et al. [96])

1. One section (4–5 μm, magnification 200×–400×) per patient is considered to be sufficient. Full sections are preferred over biopsies (in pretherapeutic neoadjuvant setting, cores can be used); currently, no validated methodology has been developed to score TILs after neoadjuvant treatment
2. TILs should be reported for the stromal compartment (% stromal TILs). The denominator used to determine the % stromal TILs is the area of stromal tissue
3. TILs should be evaluated exclusively within the borders of the invasive tumour, excluding TILs around ductal carcinoma in situ or normal lobules and zones with artefacts, necrosis, hyalinisation as well as the previous biopsy site
4. All mononuclear cells (including lymphocytes and plasma cells) should be scored, but polymorphonuclear leukocytes are excluded
5. A full assessment of average TILs in the tumour area should be used
6. It should be scored as a continuous variable that will allow categorise different thresholds and more accurate statistical analyses

*TILs* tumour-infiltrating lymphocytes

The current recommendation is that the level of TILs should not be used to withhold chemotherapy or trastuzumab therapy in TN and HER2-positive breast cancers, respectively, as the analytical validity and clinical utility of TILs remains to be firmly established. Whether TILs will be predictive of response to immunotherapeutic regimens, in particular T-cell checkpoint inhibition, has yet to be determined.

### PD-1

Programmed cell death protein (PD-1) is an immune checkpoint regulator constitutively expressed on the surface of T cells. Its major ligand, PD-L1, is expressed on the surface of TILs, antigen-presenting cells, and cancer cells including breast cancer. When PD-L1 binds to PD-1, a strong inhibitory signal is transmitted to T cells, which reduces cytokine production and suppresses T-cell proliferation. PD-L1 expression in breast cancer has been associated with poor clinical and pathological features and has been reported as preferentially expressed by basal and HER2 breast cancer [98, 99]. Therefore, it might play a role as a prognostic biomarker in the future.

The presence of tumoural PD-L1-positive TILs correlates with adverse clinic-pathological features and basal and HER2 breast cancer, but interestingly also with clinical response to PD-1 pathway blockade with anti-PD1 or anti PD-L1 targeted immunotherapy [100, 101]. Given the high costs and toxicity especially when combined with therapy, predictive biomarkers are needed. A number of ongoing trials are trying to elucidate this question.

## Conclusions

In order to plan an adequate adjuvant therapy in patients with primary breast cancer (Table 4), pathology reports must include in all cases the expression and levels of ER-alpha, PR, HER2 and Ki-67, in addition to histological grade, to assist prognosis and to establish current therapeutic options available, including hormone therapy, chemotherapy and anti-HER2 therapy.

**Table 4:** Summary of biomarkers consensus in breast cancer

Conventional markers (recommended in all patients)
ER-alpha
PR
HER2
Ki-67
Histological grade
Genetic platforms (recommended in patients with low risk of relapse)
MammaPrint^®^
Oncotype DX^®^
Prosigna^®^
EndoPredict^®^
New technologies (not recommended in routine clinical practice)
NGS Liquid biopsy and CTCs
Tumour-infiltrating lymphocytes
PD-1

*ER* estrogen receptor, *PR* progesterone receptor, *HER2* human epidermal growth factor receptor 2, *NGS* next-generation sequencing, *CTCs* circulating tumour cells

In node-negative ER-positive breast cancer patients, one of several available genetic prognostic platforms (MammaPrint^®^, Oncotype DX^®^, Prosigna^®^ or EndoPredict^®^) may be used in order to establish a prognostic category and to discuss with the patient whether adjuvant treatment may be limited to hormonal therapy.

Newer technologies including NGS, liquid biopsy, tumour-infiltrating lymphocytes or PD-1 determination are still experimental at this point.
